# Unravelling the Encapsulation of DNA and Other Biomolecules in HAp Microcalcifications of Human Breast Cancer Tissues by Raman Imaging

**DOI:** 10.3390/cancers13112658

**Published:** 2021-05-28

**Authors:** Monica Marro, Anna M. Rodríguez-Rivero, Cuauhtémoc Araujo-Andrade, Maria Teresa Fernández-Figueras, Laia Pérez-Roca, Eva Castellà, Jordi Navinés, Antonio Mariscal, Joan Francesc Julián, Pau Turon, Pablo Loza-Alvarez

**Affiliations:** 1 ICFO—Institut de Ciencies Fotoniques, The Barcelona Institute of Science and Technology, Castelldefels, 08860 Barcelona, Spain; caraujo123@yahoo.com; 2 Research and Development B. Braun Surgical, S.A.U. Ctra. de Terrassa, 121, Rubí, 08191 Barcelona, Spain; ana_maria.rodriguez_rivero@bbraun.com (A.M.R.-R.); pau.turon@bbraun.com (P.T.); 3 Campus de la UAB, Universitat Autònoma de Barcelona, Plaça Cívica, Bellaterra, 08193 Barcelona, Spain; 21127jji@gmail.com; 4 Departament de Medicina, Facultat de Medicina i Ciències de la Salut, Universitat Internacional de Catalunya, Carrer de la Immaculada, 22, 08017 Barcelona, Spain; maiteffig@gmail.com; 5 Institut de Recerca Germans Trias i Pujol (IGTP), Camí de les Escoles, s/n, 08916 Badalona, Spain; laiaproca@gmail.com (L.P.-R.); ecastella.germanstrias@gencat.cat (E.C.); drnavines@gmail.com (J.N.); mariscal.germanstrias@gencat.cat (A.M.)

**Keywords:** microcalcifications, DNA mineralization, breast cancer, tumor microenvironment, Raman spectroscopy

## Abstract

**Simple Summary:**

Although microcalcifications can be considered one of the first indicators of suspicious cancer lesions, depending on their morphology and distribution, the formation of hydroxyapatite calcifications and their relationship with malignancy remains unknown. In this work, we investigate in depth the biochemical composition of breast cancer microcalcifications, combining Raman spectroscopy imaging and advanced multivariate analysis. We demonstrate that DNA is naturally adsorbed and encapsulated inside hydroxyapatite found in breast cancer tissue. Furthermore, we also show the encapsulation of other relevant biomolecules such as lipids, proteins, cytochrome C and polysaccharides. The demonstration of the natural DNA biomineralization in cancer tissues represents an unprecedented advance in the field, as it can pave the way to understanding the role of hydroxyapatite in malignant tissues.

**Abstract:**

Microcalcifications are detected through mammography screening and, depending on their morphology and distribution (BI-RADS classification), they can be considered one of the first indicators of suspicious cancer lesions. However, the formation of hydroxyapatite (HAp) calcifications and their relationship with malignancy remains unknown. In this work, we report the most detailed three-dimensional biochemical analysis of breast cancer microcalcifications to date, combining 3D Raman spectroscopy imaging and advanced multivariate analysis in order to investigate in depth the molecular composition of HAp calcifications found in 26 breast cancer tissue biopsies. We demonstrate that DNA has been naturally adsorbed and encapsulated inside HAp microcalcifications. Furthermore, we also show the encapsulation of other relevant biomolecules in HAp calcifications, such as lipids, proteins, cytochrome C and polysaccharides. The demonstration of natural DNA biomineralization, particularly in the tumor microenvironment, represents an unprecedented advance in the field, as it can pave the way to understanding the role of HAp in malignant tissues.

## 1. Introduction

Breast cancer is by far the most frequent cancer diagnosed in women, with 2,088,849 new cases (11.6% of total diagnosed cancer) accounting for 626,679 deaths (6.6%) in 2018 [1]. However, the number of cancer survivors has been rapidly increasing due to improvements in early diagnosis and treatment efficacy (i.e., from 1989 to 2015, breast cancer death rates decreased by 39% in the US) [2,3,4]. Despite this positive trend, more efforts are needed to improve the basic understanding of the disease and the underlying cellular mechanisms in order to prevent its consequences.

Thanks to the introduction of mammographic screening programs, the early diagnosis of breast cancer has substantially improved during recent years, enabling the detection of lesions at a much earlier stage. Breast microcalcifications are often detected by means of mammographic scans, and depending on their size, morphology and distribution, they can be considered one of the first indicators of suspicious cancer lesions. Nowadays, microcalcifications are analyzed based on mammography images (BIRADS classification) and through histopathological assessments. Microcalcifications can be classified as type I or type II. Type I calcium oxalate (CaOx) microcalcifications are less frequent and are usually related to benign lesions [5,6,7,8,9]. Type II hydroxyapatite microcalcifications are frequently observed and normally associated with a worse prognosis [6,10,11,12,13]. In an effort to identify suspicious microcalcifications, several methods, such as deep learning [14], have been developed to analyze mammography scans. However, those methods are not able to provide information on the molecular composition of calcifications. Only one method, based on contrast-phase X ray tomography [15], has been reported to be able to discriminate between type I and type II calcifications in scans. However, FT-IR and Raman spectroscopy have been successfully used to investigate the adsorbed molecular composition of both types of calcifications, analyzing tissue biopsies extracted in the diagnosis phase or during surgery.

Despite multiple efforts, and many studies in the field, the mechanisms that lead to the formation of both types of microcalcifications are not completely understood, and HAp calcifications still attract the attention of researchers [10,11,12,13,14,15,16,17,18,19,20]. Traditionally, some authors focused on HAp morphology and its correlation with tumor features, whereas others focused on changes in its chemical composition (i.e., the presence of Mg^2+^ or the reduction of CO_3_^2−^ content in its lattice, which were related to a poorer prognosis) [21,22]. In contrast, CaOx calcifications have received much less attention, even though their cytotoxicity and risk of carcinogenicity have been described [23,24]. O’Grady and Morgan (2018) recently reviewed breast cancer microcalcifications, covering a wide range of topics, from pathophysiology to diagnosis and prognosis [25]. However, there are few publications that have studied the biochemical composition of breast cancer calcifications and none of them describing the tridimensional structure [16,17,26,27,28]. Therefore, a method that is capable of investigating the biochemical composition in detail, along with the spatial distribution of biomolecules inside the calcification and the surrounding tissue, is a key objective in order to understand the formation of HAp calcifications and their associated worse prognosis.

Raman spectroscopy is an excellent technique for the detailed analysis of the biochemical content of tissues because of its high specificity, non-destructive nature and label-free approach [29,30,31]. Raman spectroscopy utilizes inelastic light scattering to extract vibrational molecular fingerprints of molecules present in the samples. It enables the simultaneously study of multiple molecular components present in a sample. Such an approach is not possible with other techniques such as fluorescence staining, which require the use of previously selected labels, making the study of several molecules in the same sample at the same time difficult. In addition, DNA fluorescent labels, such as DAPI, might not efficiently penetrate microcalcifications and the fluorescence bands may be shifted due to the interaction of the label with the microcalcification [32,33]. Consequently, the localization and identification of the molecular content in the microcalcification might not be feasible or sufficiently accurate with other techniques. In previous studies, Raman spectroscopy has been demonstrated as an optimal technique to study breast tissue microcalcifications, because the Raman spectra of different calcium salts exhibit well defined signals with very distinct features. First, Haka et al. [6] showed that it was possible to identify different chemical compositions in benign and malignant breast microcalcifications using a single acquisition in the calcified region. Then, Stone et al. [7] successfully detected microcalcifications deeply buried in tissues by means of spatially offset Raman spectroscopy (SORS). Baker et al. reported a decrease in the carbonate content of HAp from benign to malignant calcifications using infrared spectroscopy [17]. Recently, it was shown that different inorganic compositions of microcalcifications correlate with histopathologic features [28]. Despite these efforts, the detailed tridimensional molecular composition adsorbed or encapsulated in such microcalcifications has never been studied. Specifically, the distribution of organic molecules in three dimensions has not yet been reported. In addition, previous Raman spectroscopic studies have considered a low number of calcifications and the analysis of their relationship with invasive tissues has been almost absent. Furthermore, due to the rich information collected by Raman spectroscopy, the biological interpretation of spectra has become complex, and many research works have not sufficiently analyzed the molecular information available. Recently, the use of advanced multivariate analysis methods has enabled the rigorous study of complex biological Raman spectra in detail [30,31].

**Figure 1:**
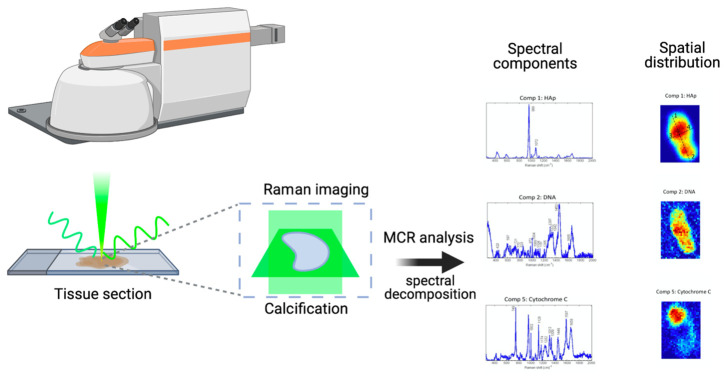
Workflow and experimental design: in this paper, we demonstrate a methodology that is capable of investigating the biochemical composition of HAp calcifications in human breast cancer tissues. First, we perform Raman imaging of breast cancer tissue sections (*n* = 26 patients), acquiring 2D images in perpendicular directions (xy, xz and yz) in order to retrieve 3D molecular information. Second, we use an advanced multivariate analysis method, multivariate curve resolution (MCR) to extract otherwise inaccessible molecular information encoded in the Raman spectra. Specifically, we decompose several spectral components, corresponding to organic molecules and their associated 3D molecular spatial distribution in HAp microcalcifications. Through this method, we show the presence and 3D spatial distribution of several molecular components (DNA, lipids, proteins, cytochrome C and polysaccharides) in the HAp calcifications extracted from cancer tissues.

In this paper, we describe a methodology that enables the investigation of the usual biochemical composition of HAp calcifications observed in human breast cancer tissues (Figure 1). We report the most detailed tridimensional study of breast cancer HAp microcalcifications to date, combining molecular Raman imaging of breast cancer tissue sections (*n* = 26 patients) with an advanced multivariate analysis method, multivariate curve resolution (MCR). The use of advanced data analysis enables the extraction of otherwise inaccessible molecular information encoded in the Raman spectra. Specifically, we focus on the study of the 3D molecular distribution of organic molecules in HAp microcalcifications. We show, for the first time, the spatial distribution of several molecular components (DNA, lipids, proteins, cytochrome C and polysaccharides) in the HAp calcifications extracted from a tumor microenvironment. Furthermore, this study is grounded on the most extensive Raman spectra analysis performed on breast tissue calcifications (more than 10,400 spectra).

Specifically, we show that DNA is encapsulated and adsorbed naturally in the HAp present in invasive cancer tissues. The natural biomineralization of DNA in breast cancer tissues would enable the preservation of this molecule for long periods of time, protecting it from enzymatic degradation [26,34,35,36,37,38]. This finding represents a major advance in the field, as it can pave the way to understanding the formation and the role of HAp calcifications in malignant tissues.

## 2. Materials and Methods

### 2.1. Breast Tumor Samples

Samples and data from patients included in this study were provided by the IGTP-HUGTP Biobank, integrated in the Spanish National Biobank Network of Instituto de Salud Carlos III (PT13/0010/0009) and the Tumor Bank Network of Catalonia. The samples were processed following standard operating procedures with the appropriate approval of the Ethical and Scientific Committees (CCEBB IGTP-HUGTiP Request: BB14004 and evaluation Ref: BB-C-1402). Selected tissue specimens of the tumors were embedded in an optimal cutting temperature (OCT) compound and frozen in isopentane using a Bright Clini-RF freezer for 30 min after extraction.

Tissue sections from freshly excised human breast tumors (*n* = 26 patients) were studied (Table 1). Samples were diagnosed as infiltrating ductal and lobular carcinomas and microcalcifications were observed in previous mammography screenings.

### 2.2. Raman Spectroscopy

Thawed tissue sections of 5 μm in thickness were measured in quartz slides after conditioning at room temperature for 30 min. An inVia Renishaw Raman microscope was used. A visible 532-nm laser excitation with 10 mW and a 50× objective lens was used. Raman images were acquired with 1 s acquisition time and 1-µm pixel size. For in-depth area acquisitions, a 0.5-µm pixel size was selected in the z direction.

### 2.3. Multivariate Analysis

To determine the chemical composition for the Raman images, we used multivariate curve resolution (MCR). For MCR analysis, a Matlab toolbox was used: PLS toolbox (from Eigenvector Research). Raman spectra were pre-processed before multivariate analysis. A background correction was performed using the baseline correction method; asymmetric least squares smoothing and an extended multiplicative scattering correction (EMSC) for multiplicative effects was applied [39].

First, an exploration of the spectral dataset was performed using principal component analysis (PCA). The initial number of components selected was the number in which the cumulative variance explained was more than 99%. Two independent analyses (PCA and MCR) were performed on the same dataset, which contained the spectra of the breast cancer tissues in rows. For the MCR-ALS algorithm, the protocol followed was as follows. First, the initial estimates were selected using the SIMPLISMA algorithm, which selects the purest rows (spectra) in the dataset; second, the iterative least squares calculation began with the addition of constraints (non-negativity of spectra and concentration matrices). Finally, the quality parameters from the MCR model were calculated—Lack of fit: $LOF=100\sqrt{\frac{\sum_{i=1}^{n}\sum_{j=1}^{m}e_{i,j}^{2}}{\sum_{i=1}^{n}\sum_{j=1}^{m}d_{i,j}^{2}}}$ and the parameter: $R^{2}=100\frac{\sum_{i=1}^{n}\sum_{j=1}^{m}d_{i,j}^{2}-\sum_{i=1}^{n}\sum_{j=1}^{m}e_{i,j}^{2}}{\sum_{i=1}^{n}\sum_{j=1}^{m}d_{i,j}^{2}}$ where $e_{i,j}=d_{i,j}-{\hat{d}}_{i,j}$ and $d_{i,j}$ is the original data matrix and ${\hat{d}}_{i,j}$ is calculated by means of the MCR-ALS algorithm.

### 2.4. Breast Tumor Sample Staining for Confocal Microscopy Imaging 

OCT-fixed tissue section samples were rehydrated by immersing them in PBS for 15 min at room temperature and then staining them with DAPI.

### 2.5. In Vitro Synthesis of DNA-HAp Particles

Suspensions of HAp particles (5 mg·mL^−1^) were prepared, following the procedure described elsewhere [34], and were subsequently sonicated to enhance the dispersion. DNA was extracted from 4T1-luc2 cells (Perkin Elmer, 124087). DNA-HAp complexes were formed to reach 1% DNA, on HAp *w*/*w*. DNA-HAp mixtures were incubated for 90 min at 37 °C and 200 rpm, shaking them with a vortex every 30 min. Complexes were separated from the solution by centrifugation at 10,000 rpm for 10 min. Sediments were re-suspended in 10 mL of sterile water.

## 3. Results

### 3.1. Molecular Raman Imaging and Advanced Multivariate Analysis of Tissue Calcifications

Revealing the presence and spatial distribution of biomolecules in microcalcifications might contribute to understanding their role in breast cancer tissue. In this paper, we applied an advanced multivariate analysis approach for the first time to Raman images to obtain new insights about the biomolecular content of breast cancer microcalcifications.

Raman spectral images were acquired from tissue sections of freshly excised human breast tumors (*n* = 26 patients). The measured samples were diagnosed as infiltrating ductal and lobular carcinomas. Microcalcifications were observed in previous mammographic scans. In Table 1, a detailed summary of the analyzed samples is presented.

**Figure 2:**
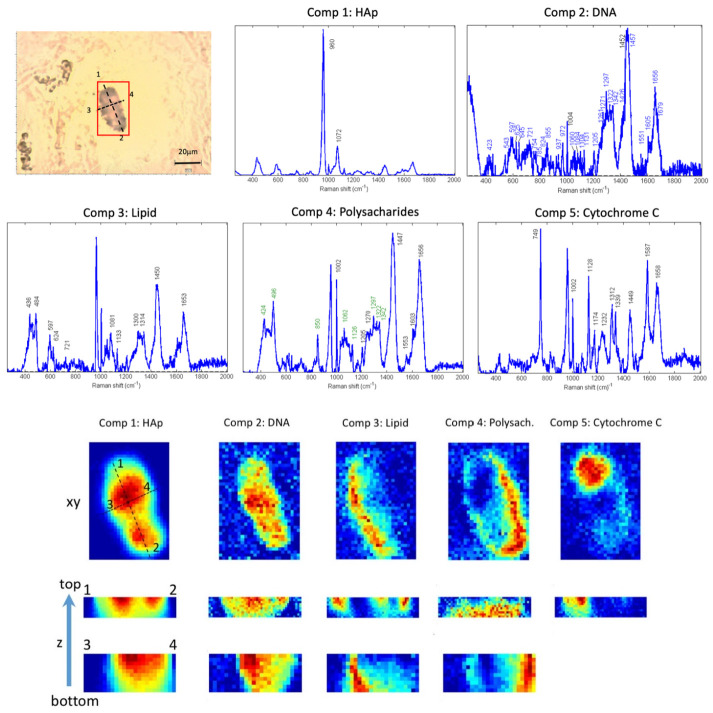
Raman spectral images show the presence of different molecular components in HAp tissue calcifications. Top left: Bright field image of a breast tissue section with a HAp calcification highlighted in the region of interest (red box). Raman images were taken from this area. Using the MCR algorithm, several molecular components were deconvolved from the spectra. In the first and second rows, the spectral signatures of the deconvolved molecular component are shown. For each component, its corresponding abundance maps are plotted in the bottom half of the Figure. The last two rows are perpendicular sections imaged to show the distribution of the molecules in depth inside the tissue. This microcalcification was found in tissue number 20 of Table 1. The assignment of bands for the different components was performed based on our constructed database and the literature [40,41,42,43].

We used MCR to fully extract the complex biomolecular information contained in the Raman spectra. As a result, we were able to deconvolve multiple molecular spectral signatures that could be assigned to specific molecular components present in the studied calcifications. In addition, associated with those molecular spectral fingerprints, we retrieved the related concentration maps, showing the distribution of the molecular components along the area and volume of the sample studied (Figure 2). Therefore, through the use of this method, we provide information not only about the molecular composition but also about the spatial distribution of molecules contained in the tissue calcifications. As we studied Raman images in the tissue section plane and two perpendicular 2D sections, our multivariate analysis methodology enabled us to understand if a certain molecular component was located inside, on the surface of, or outside the microcalcfications. The results for a HAp calcification are shown in Figure 2.

A summary of the analyzed tissues can be found in Table 1. For each calcification, information about the size, shape, localization and molecular content is shown. We gathered the information about the size and the shape because the first radiological diagnosis is based not only on the presence of calcifications but also on the shape and distribution of the calcifications in the tissue, providing hints about the aggressiveness of the lesion.

**Figure 3:**
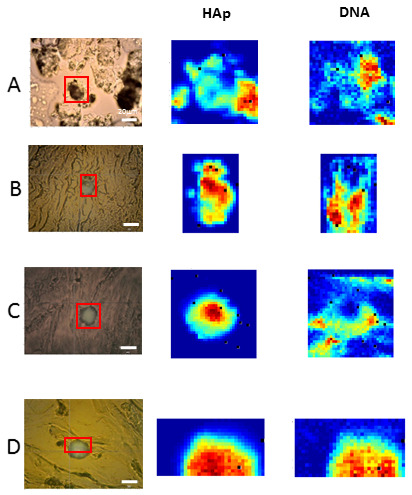
Raman spectral images of different breast cancer tissue sections showed the presence of DNA in HAp calcifications. First column: Bright field image of the breast tissue section imaged, with a HAp calcification highlighted in the region of interest (red box). Raman images were obtained from this area. Using the MCR algorithm, several molecular components were deconvolved from the spectra. The abundance maps of HAp and DNA molecular components are plotted in the second and third columns. Tissues (**A**–**D**) correspond to tissues 20, 22, 1 and 9 in Table 1.

We found HAp calcifications in 24 out of the 26 tissues studied (92.3%). In Figure 2, the results of an HAp calcification are shown (from tissue number 20 in Table 1). In Figure 2 a bright field image of the microcalcification is displayed, highlighting the area in which the Raman map was acquired. In total, five molecular spectral profiles were decomposed using the MCR-ALS algorithm. The associated abundance maps describing the distribution of the different molecular components in the area of the Raman image are shown below. Two perpendicular z-section Raman images were obtained to visualize the molecular content inside calcifications (last two rows of Figure 1). Component 1 has the spectral profile of HAp, defining the area of the calcification which correlates with the bright field image. The bands that confirm the assignment of this component to HAp are indicated in the spectra and are in agreement with the literature [22]. Component 2 contains a rich biological spectrum, having several bands in the fingerprint region, and is distributed inside the HAp calcification (plane (xy) and in depth (z) images from concentration maps). Components 3, 4 and 5 also have a rich spectral profile with bands that can be assigned to biological molecules. Components 2 and 5 are detected inside the tissue HAp calcification. However, Components 3 and 4 are found at the edges of the HAp calcification, indicating that they are adsorbed on the surface of the calcification. Such a pattern is repeated for almost all the HAp calcifications studied (24 out of 26, see Table 1), presenting biological content inside or adsorbed in the surface of the HAp calcifications (Figure 3). A further in-depth molecular assignment of the deconvolved MCR components is discussed in Section 3.2 and Section 3.3. These results demonstrate that important biomolecules biomineralize in HAp calcifications. The observation that HAp mineralizes biological material could be related to its worse prognosis. Therefore, in the next section we investigate in depth the assignments of the biomolecular components found in HAp.

### 3.2. Study of DNA Mineralization in HAp Microcalcifications

Recent studies demonstrate that nucleic acids can be biomineralized and remain functional for long times [26], adsorbed or encapsulated in the mineral. Therefore, biomineralized DNA found in cancer tissues might have important implications, as biomineralized DNA is a hybrid system known for acting as a non-viral vector of transfection that has the ability to transfect to in vitro cells [38,44]. For this reason, we focused on studying in detail the presence and 3D distribution of DNA in microcalcifications.

#### 3.2.1. Reference Raman Spectral Databases and Literature

**Figure 4:**
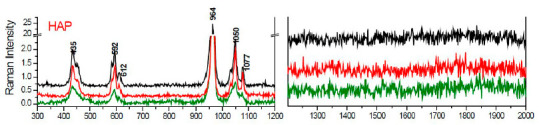
Raman spectrum of HAp synthetized in the laboratory.

To characterize and study the presence of DNA and other molecules in HAp calcifications, we measured the spectra of HAp crystals synthesized in the laboratory (Figure 4) and created a spectral database of bands assigned to DNA and other biomolecules that may be present in cancer tissues, based on the reports shown in literature [40,41,42,43]. HAp crystals exhibit a main band at 964 cm^−1^ and other smaller bands at 435, 592, 1050 and 1077 cm^−1^ (Figure 4). These observed bands are compatible with the ones reported in the literature [40]. The Raman spectra of DNA have been broadly studied and characterized and its main Raman bands have been reported [40,43].

#### 3.2.2. Identification of DNA in HAp Calcifications Found in Breast Cancer Tissues

The spectral features obtained from the synthetic HAp crystals and the Raman databases of biomolecules found in the literature were used as a reference to study the Raman images acquired from the excised breast cancer samples. In Figure 2, the different spectral profiles of the components extracted by MCR-ALS algorithm are shown, specifying the position of the main Raman bands.

In Table 2, we summarize the bands and assignments of molecular component number 2 extracted from the MCR analysis of HAp calcification, shown in Figure 2. Assignments were based on the literature [40,43]. More than 90% of the spectral band positions in component 2 correspond to the Raman bands also found in DNA.

Analyzing the distribution of Component 2 in the Raman imaging abundance maps (Figure 2), we can observe that DNA is present inside the HAp calcifications, as its spectra coincide with the spatial distribution of the HAp spectral component (Component 1). Z-sections along lines 1–2 and 3–4 confirm the presence of DNA inside the region of the HAp calcification. Therefore, we conclude that DNA is present and surrounded by HAp crystals in natural HAp calcifications found in breast cancer tissues.

The majority (92.3%) of HAp calcifications studied in breast cancer tissues contained DNA. Specifically, a DNA component was found in 91.3% of samples (23 patients) for infiltrating ductal carcinoma and 100% of samples (three patients) for lobular carcinoma. All details are shown in Table 1. In some cases, the presence of DNA was found more abundantly at the border of the microcalcifications, which indicates that DNA cannot only be encapsulated within, but must also be adhered to the surface of, the HAp calcification. Raman images of other analyzed breast cancer tissue sections are shown in Figure 3.

#### 3.2.3. Fluorescence Staining of Microcalcifications with DAPI

**Figure 5:**
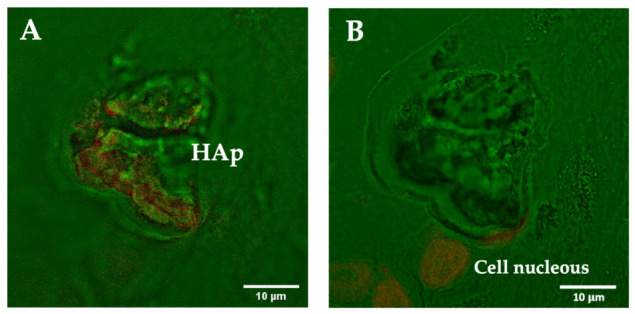
Confocal microscopy images of a breast cancer tissue section containing a HAp calcification at different depths: (**A**) image focused on the plane of the calcification, and (**B**) image focused on the plane of the surrounding cells in the tissue. DNA staining was performed with DAPI. Bright field (green) and DAPI (red). DAPI staining indicates the presence of DNA inside HAp calcifications and enables the visualization of the nucleus of the surrounding cells.

To confirm the presence of DNA with a different technique, we stained tissue sections with DAPI. Staining has several complications and limitations because dyes might not penetrate in an effective way inside the calcifications. For this reason, we used DAPI as a complementary technique to our work. Images of HAp calcifications were acquired at different depths (Figure 5). Figure 5A shows a HAp calcification stained with DAPI that has an elongated region in one extreme, which indicates the presence of DNA in this area. Going deeper into the sample, the end of the HAp calcification is encountered, and the plane of the surrounding tissue can be visualized (Figure 5B). This area contains cells, and we can observe the nucleus stained with DAPI, which confirms that the correct staining procedure was used. Although fluorescence staining results are only reliable in mapping exposed DNA on the surface but not in the interior (because of the issues related to the penetration of the dye in the calcification and the fluorescence shifts), these results support the Raman results indicating that DNA is trapped inside HAp.

### 3.3. Raman Images Reveal the Presence and Distribution of Biomolecules Inside HAp Calcifications

Raman images in different optical sections (in x, y, z) confirmed that cancer tissue HAp calcifications contain several molecular components (Figure 2). Apart from the DNA, we found other molecular components present in the HAp tissue calcifications. The assignments of the bands of each spectral profile in Figure 2 are summarized in Table 3 and were assessed based on the literature [40,41,42,43].

First, Component 3 was extracted, presenting Raman bands at 436, 484, 597, 624, 721, 1081, 1133, 1300, 1314, 1450 and 1653 cm^−1^. The majority of the Raman bands can be assigned to lipids [40,41,43]. According to the abundance map, this molecular component is present at the borders of the HAp (Figure 2). The two perpendicular z-section Raman images confirmed its localization at the surface of the calcification.

Second, Component 4 presents Raman bands at 424, 496, 850, 1002, 1062, 1126, 1205, 1270, 1297, 1322, 1342, 1447, 1553, 1603 and 1656 cm^−1^. These bands can be assigned to polysaccharides (labeled in green) and proteins [40,43]. Component 4 is also present at the borders of the HAp calcification (Figure 2).

Finally, Component 5 presents bands (749, 1002, 1128, 1174, 1232, 1312, 1339, 1449, 1587 and 1658 cm^−1^) that can be assigned to cytochrome C [42] (Figure 2). Thanks to the abundance maps in x-y and z we can confirm that this molecular component is located inside the HAp calcification.

These molecular components are common in most cells and human tissues. With these results, we are able to reveal the natural encapsulation or adhesion of relevant biological molecules in HAp microcalcifications of breast cancer tissues. The molecular information, together with the morphological distribution of the components in the calcification, could provide hints on how the calcification was initiated, indicating that many cellular and tissue molecular components might be biomineralized during calcification.

## 4. Discussion

Microcalcifications are key features observed in breast mammography and, depending on the BIRADS classification method (based on morphological and distribution characteristics like shape and cluster organization), they are associated with a different degree of malignancy. However, the formation of HAp calcification in cancer tissues and its association with a worse prognosis is still not well understood. To address these questions, it is key to develop a method that is capable of investigating in detail the biochemical composition and the spatial distribution of biomolecules in the calcification and the surrounding tissue in a label-free approach. Raman spectroscopy has been shown to be a promising tool to characterize microcalcifications [5,6,7]. Specifically, a recent paper [28] showed that Raman spectroscopy of calcifications correlates with histopathology, while being more rapid and objective. Furthermore, RS would be able to be used for in depth measurements using SORS [7], or used in needle biopsies for the non-invasive future assessment of calcifications. However, despite the number of the studies in the field, there is a lack of in-depth chemical analysis of the content of microcalcifications exploiting the rich molecular information contained in Raman spectra.

In this study, we combined Raman imaging of breast cancer microcalcifications with advanced multivariate analysis (MCR-ALS). Such an approach enabled us to provide an in-depth analysis of the composition and distribution of different molecular components inside tissue calcifications, extracting meaningful biological information. Some previous studies have reported a low number of samples studied. Furthermore, most of them studied individual spectra at different sites on the calcification. Only two studies have recently used Raman imaging [27,28]. However, in [27], only one image was studied for exploration purposes, and in [28] the image spectra of calcifications were averaged, thus losing the spatial information. In general, in all studies, multivariate analysis has been used to classify Raman spectra. Overall, all articles lack a multivariate analysis technique to study the rich molecular information that is contained in the Raman spectra in detail. In our work, we go a step further, extracting biological information from the Raman data.

The Raman spectra of biomolecules such as DNA, proteins, lipids, etc., have been largely characterized and reported [40,41,42,43]. However, biological samples such as tissues are complex because they contain mixtures of multiple biomolecules. In order to extract all the molecular information encoded in the Raman spectral images, we used MCR-ALS [30,31]. This multivariate algorithm enables the extraction of multiple components that are present in the sample studied. For each component, a molecular Raman spectral shape is retrieved and associated with the distribution or concentration of the molecular component along the spectra acquired. In this case, as we acquired images, we were able to reconstruct the 3D molecular distribution of the components along the microcalcification studied. This algorithm is of particular interest for biomedical analysis because relevant information about the identity of the molecules encrusted in those calcifications can be extracted. First, the algorithm does not require previous molecular information about the sample, and it is an unsupervised method. Second, the extracted spectral signatures of the different components could be assigned to Raman spectra of biological molecules. Therefore, by employing the MCR-ALS algorithm, we were able to retrieve spectral components that could be assigned to pure molecules or a combination of molecules that were present in the sample in the same locations. Thus, this enabled us to study the distribution and localization of different molecular components in the microcalcifications studied. Furthermore, as we obtained Raman images and two respective perpendicular z-sections, we were able to localize the different molecular components and study whether the molecules were located inside the calcification, adsorbed on its surface, or if they could be found outside it (in the surrounding tissue). Therefore, our methodology and work contribute novel results, adding important information to the state of the art, revealing information that is otherwise inaccessible with other techniques.

Raman spectroscopy studies on breast cancer microcalcifications have focused on the study of the inorganic content of calcifications. Only in few cases were organic components reported, but without an in-depth analysis [27,28]. However, our advanced multivariate methodology enabled us to concentrate on extracting for the first time different biological molecular components present in the breast microcalcifications. Therefore, the focus of our study is an in-depth analysis of biological material trapped in microcalcifications and its distribution in the calcification.

The first relevant result of this work is that HAp calcifications contain several molecular components that can be assigned to biological molecules distributed inside or adsorbed in the calcifications’ volume. First, we observed that DNA is contained inside or adsorbed on the surface of HAp calcifications in a high percentage of cases (92.3%). The demonstration of the biomineralization of DNA in HAp is important because (a) recent studies demonstrated that DNA could be preserved in HAp for long periods of time [26], and (b) DNA can be transferred to a different cell after some time [38]. Therefore, these results represent a large step toward a deeper understanding of the formation and role of HAp calcifications and their association with a worse prognosis.

Another important finding that we report in this work is that HAp calcifications contain other significant biological molecules inside them or adsorbed on their surface. Molecules such as cytochrome C, lipids and polysaccharides were identified. This suggests that HAp calcifications could be formed as a result of specific cellular processes, in particular in a non-regulated cell death, where the uncontrolled release of calcium and phosphate ions in combination with DNA occurs and leads to the formation of necrotic microcalcifications. On the other hand, studies performed on tissue samples and in vitro on cultured cells demonstrated that microcalcification formation is a cell active process, influenced by the microenvironment and by the overexpression of bone matrix proteins (i.e., osteonectin and osteopontin) [16,28,45]. Both studies reported that active processes of microcalcification formation are significantly more represented in cases of malignancy. In parallel, a retrospective study on patients referred for needle-guided biopsy reported that the formation of new microcalcifications significantly correlates with a high probability of ductal invasive carcinoma [46]. According to [28], the crystallinity and homogeneity of malignant microcalcifications that they found in their study could originate from a faster and more active process, stimulated by cancer and by its microenvironment. The presence of cytochrome C is considered to be a sign of cell apoptosis [47]. Cytochrome C is usually found in the extracellular space as a result of pathological conditions in which cell death occurs. The fact that cytochrome C is associated with HAp calcifications reinforces the hypothesis that the calcifications of HAp containing DNA are formed when cell death processes occur in the tumor. Thus, the Raman molecular fingerprint found in this study suggests that a pathological process is behind the origin of the natural biomineralization of DNA in tumor HAp calcifications.

Apart from the HAp calcifications measured, six different CaOx calcifications were analyzed, found in one infiltrating lobular carcinoma tissue sample (Figure S1). No traces of DNA were found inside the CaOx calcifications. Although it will be necessary to increase the number of samples analyzed, these data are consistent with the simulation results published recently [26] showing that the tendency to adsorb and especially encapsulate DNA is much smaller for CaOx than for HAp, particularly because Mg^2+^ hinders the adsorption of DNA on CaOx.

Raman imaging, together with the multivariate analysis methodology presented here, represents an important step forward in the field because new biological information can be obtained without requiring initial information about the composition of the sample. Furthermore, there is no need to use specifically developed immuno-labels to target the relevant molecules. The study of calcifications with immunofluorescence has some hurdles that must be considered in order to create a 3D mapping of the calcification. First, immunolabels may not penetrate the outer layer of the calcification, thus failing to reach certain parts of the biomineralized materials. Second, immunofluorescence requires a priori knowledge of the biological molecules existing in the sample in order to stain the tissue and image it. In this case, no prior information was known. Third, there is a limited number of immune labels that can be imaged, obtaining therefore only partial information. However, with our methodology, we demonstrated that tridimensional information on several biological molecular components could be extracted without the need for previous knowledge, minimizing the risk of perturbing the sample or the need to add chemicals that modify it. Therefore, new and otherwise inaccessible molecular information has been obtained, progressing the understanding of the association of HAp calcifications with malignancy and their role in cancer tissue.

## 5. Conclusions

In this paper we have presented a methodology that is capable of investigating the biochemical composition of HAp calcifications in human breast cancer tissue. We report the most detailed study on the biochemical analysis of breast cancer HAp microcalcifications to date, combining molecular Raman imaging of breast cancer tissue sections (*n* = 26 patients) with an advanced multivariate analysis method, multivariate curve resolution (MCR). The use of advanced data analysis has enabled the extraction of otherwise inaccessible molecular information encoded in the Raman spectra. Specifically, we focused on the detailed study of the 3D molecular distribution of organic molecules in HAp microcalcifications. We have shown for the first time the presence and spatial distribution of several molecular components (DNA, lipids, proteins, cytochrome C and polysaccharides) in the HAp calcifications extracted from living cancer tissue. Specifically, we have shown that DNA is encapsulated or adsorbed naturally in HAp calcifications present in invasive cancer tissues. The natural biomineralization of tumoral DNA in breast cancer tissues could enable the preservation of this molecule for long periods of time. This finding represents a substantial advance in the field as it can pave the way toward understanding the formation and the role of HAp calcifications in malignant tissues.

## Figures and Tables

**Table 1:** Summary of the microcalcifications measured by Raman spectroscopy found in the human breast cancer tissue sections analyzed. For each calcification, we specify the diagnosis, the type (Oxalate or HAp), content of DNA (according to Raman spectroscopy results), size, shape, and localization.

	Diagnosis	Oxalate	HAp	DNA	Size ^a^	Shape ^b^	Localisation ^c^
1	Infiltrating ductal carcinoma (IDC)	No	Yes	Yes	m, D = 20 µm	S	t
2	Infiltrating ductal carcinoma (IDC)	No	Yes	Yes	m, D = 20 µm	R	t
3	Infiltrating ductal carcinoma (IDC)	No	Yes	No	m, D = 10 µm	S	t
3	Infiltrating ductal carcinoma (IDC)	No	Yes	Yes	m	R	t
4	Infiltrating ductal carcinoma (IDC)	No	Yes	Yes	m, D = 20 µm	R	d
5	Infiltrating ductal carcinoma (IDC)	No	Yes	Yes	m, D = 20 µm	S	t
5	Infiltrating ductal carcinoma (IDC)	No	Yes	Yes	m, 30 × 20 µm	R	t
6	Infiltrating ductal carcinoma (IDC)	No	No	No	-	-	-
7	Infiltrating ductal carcinoma (IDC)	No	Yes	Yes	m, D = 10 µm	R	t
8	Infiltrating ductal carcinoma (IDC)	No	No	No	-	-	-
9	Infiltrating ductal carcinoma (IDC)	No	Yes	Yes	m, 25 × 15 µm	R	t
9	Infiltrating ductal carcinoma (IDC)	No	Yes	Yes	m, 20 µm	S	t
10	Infiltrating ductal carcinoma (IDC), multifocal	No	Yes	Yes	m, 30 µm	R	t
11	Infiltrating ductal carcinoma (IDC)	No	Yes	Yes	m	R	t
12	Infiltrating ductal carcinoma (IDC)	No	No	-	-	-	-
13	Infiltrating ductal carcinoma (IDC)	No	Yes	No	b	R	t
14	Infiltrating ductal carcinoma (IDC)	No	Yes	Yes (nc)	S	R	t
15	Infiltrating ductal carcinoma (IDC)	No	Yes	Yes	b	R	t
16	Infiltrating ductal carcinoma (IDC)	No	Yes	Yes	m, 100 µm	R	d
17	Infiltrating ductal carcinoma (IDC)	No	Yes	Yes	m, 40 × 50 µm	R	t
18	Infiltrating ductal carcinoma (IDC)	No	Yes	Yes	s, D = 7 µm	S	t
19	Infiltrating ductal carcinoma (IDC)	No	Yes	Yes	m	R	t
20	Infiltrating ductal carcinoma (IDC)	No	Yes	Yes	m	R	d
21	Infiltrating ductal carcinoma (IDC)	No	Yes	Yes	m	R	d
22	Infiltrating ductal carcinoma (IDC)	No	Yes	Yes	m, 20 × 10 µm	R	t
23	Infiltrating ductal carcinoma (IDC)	No	Yes	Yes	b	R	t
24	Infiltrating lobular carcinoma (ILC)	No	Yes	Yes	m, D = 20 µm	R	t
25	Infiltrating lobular carcinoma (ILC)	Yes	No	No	s	S	t
25	Infiltrating lobular carcinoma (ILC)	No	Yes	Yes	m	R	t
26	Infiltrating lobular carcinoma (ILC)	No	Yes	Yes	m	R	t

^a^ Size: small, s (<10 µm); medium, m (10 µm < m < 100 µm); big, b (>100 µm). ^b^ Shape: rock, R; sphere, S; needle, N. ^c^ Localization: duct, d; tissue, t.

**Table 2:** Bands and assignments of molecular component number 2, extracted from MCR analysis of HAp calcification shown in Figure 2. Assignments were based on the literature [40,43].

Component 2: DNA			
Band/cm^−1^	Assignment	Band/cm^−1^	Assignment
1679	T, DNA base	1084	Phosphodiester groups in nucleic acids
1656	T, G, C (ring breathing modes of the DNA/RNA bases); amide I (protein); C=C lipids	1060	PO_2_-stretching of DNA
1605	Cytosine (NH2)	1004	Phenylalanine
1551	Guanine	972	T, ribose, Phosphate monoester groups of phosphorylated proteins and cellular nucleic acids
1457	A, C, T DNA bases	937	G, A DNA bases
1452	CH_2_ CH_3_ deformation	855	Phosphate group
1426	Deoxyribose (B, Z marker)	824	O-P-O stretch DNA
1342	A, G DNA bases	785	U, T, C, O-P-O
1322	G, DNA base	754	T, DNA
1297	C, palmitic acid, CH_2_	721	A, DNA base
1271	C, G DNA bases	645	G DNA base
1261	A, G, C DNA bases	625	A, T DNA bases
1205	A, T, ring breathing mode of DNA bases	597	C DNA base
1131	A DNA base	543	C DNA base
1104	O-P-O backbone stretch DNA, U	423	T, U DNA bases

**Table 3:** Bands and assignments of molecular spectral components extracted from MCR analysis of the Raman image of the tissue HAp calcification shown in Figure 2. Assignments were based on the literature [40,41,42,43].

Component 3: Lipid		Component 4: Polysaccharides and Proteins		Component 5: Cytochrome C	
Band/cm^−1^	Assignment	Band/cm^−1^	Assignment	Band/cm^−1^	Assignment
436	Cholesterol, cholesterol ester	424	Polysaccharide	749	Cytochrome C
597	Phosphatidylinositol	496	Glycogen	1002	Phenylalanine
624	Cholesterol ester	850	Polysaccharide	1128	Cytochrome C
721	Phosphatidylcholine, Sphingomyelin	1002	Phenylalanine	1174	CH tyrosine phenylalanine
1081	Phosphatidylcholine, membrane lipids	1062	C-C carbohydrates	1232	Amide III
1133	Membrane lipids (PC, PE, SM)	1126	Disaccharides, glucose, sucrose, C-O stretching carbohydrates	1312	Cytochrome C
1300	Membrane lipids (PC, PE)	1205	Amide III proteins	1339	tryptophan
1314	CH3CH2 twisting of lipid	1270	Amide III proteins	1449	C-H proteins
1450	CH (lipids and proteins)	1297	CH2 deformation	1587	Cytochrome C
1653	C=C lipids, proteins	1322	CH deformation proteins	1658	Amide I proteins
		1342	CH deformation protein and carbohydrates		
		1447	CH2 deformation proteins		
		1553	Tryptophan, Amide II		
		1603	Phenylalanine		
		1656	Amide I proteins		

## Data Availability

Data are contained within the article or supplementary material.

## Supplementary Materials

The following are available online at https://www.mdpi.com/article/10.3390/cancers13112658/s1, Figure S1: Raman image of an oxalate calcification.
