# Supplementary material for: Unravelling the Encapsulation of DNA and Other Biomolecules in HAp Microcalcifications of Human Breast Cancer Tissues by Raman Imaging
Source: Cancers (Basel). 2021 May 28;13(11):2658. doi: 10.3390/cancers13112658 (PMC8198780; doi:10.3390/cancers13112658)
Supplement: Supplementary file 1 [file cancers-13-02658-s001.zip › cancers-1223557-supplementary-final.pdf]

Unravelling the Encapsulation of DNA and Other biomolecules in HAp Microcalcifications of Human Breast Cancer Tissues by Raman Imaging

Monica Marro, Anna M. Rodríguez-Rivero, Cuauhtémoc Araujo-Andrade, Maria Teresa Fernández-Figueras, Laia Pérez-Roca, Eva Castellà, Jordi Navinés, Antonio Mariscal, Joan Francesc Julián, Pau Turon and Pablo Loza-Alvarez

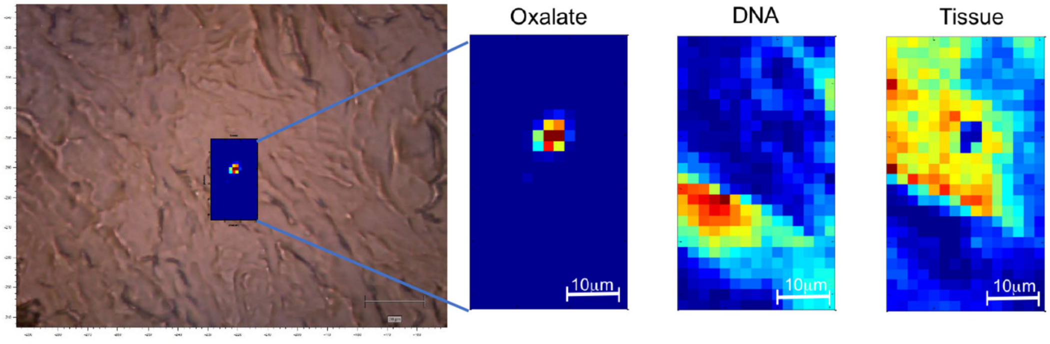

Figure S1. Raman image of an oxalate calcification.
